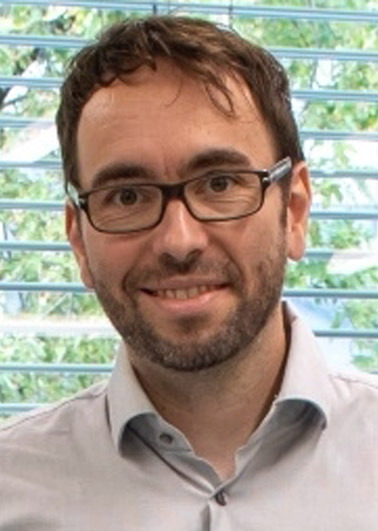# Derivatives of Natural Organocatalytic Cofactors and Artificial Organocatalytic Cofactors as Catalysts in Enzymes

**DOI:** 10.1002/cbic.202100599

**Published:** 2022-04-01

**Authors:** Horst Lechner, Gustav Oberdorfer

**Affiliations:** ^1^ Graz University of Technology Institute of Biochemistry Petersgasse 10–12/II 8010 Graz Austria

**Keywords:** biocatalysis, cofactors, enzyme engineering, organocatalysis

## Abstract

Catalytically active non‐metal cofactors in enzymes carry out a variety of different reactions. The efforts to develop derivatives of naturally occurring cofactors such as flavins or pyridoxal phosphate and the advances to design new, non‐natural cofactors are reviewed here. We report the status quo for enzymes harboring organocatalysts as derivatives of natural cofactors or as artificial ones and their application in the asymmetric synthesis of various compounds.

## Graphical abstract

**Organocatalysts meet proteins**: Enzymatic activities by natural organocatalytic cofactors, non‐natural derivatives of them and artificial cofactors are reviewed.

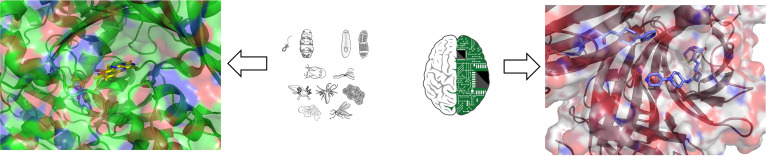

## 1 Introduction

“*The catalysis with small organic molecules, where an inorganic element is not part of the active principle*”^[1]^ is a very descriptive definition of organocatalysis. The concept of organocatalysis has been known for more than 100 years. But it has only gained attraction as research field since the year 2000, when List, Lerner and Barbas showed the application of L‐proline as catalyst for an asymmetric aldol reaction^[2]^ and Ahrent, Borths and MacMillan used chiral imidazolidinones for an enantioselective Diels‐Alder reaction.^[3]^ Since then, the number of reported catalysts and applications has grown immensely. For their efforts in this field, List and MacMillan were awarded with the Nobel Prize in Chemistry in 2021. Moreover, enantioselective organocatalysis was selected as one of ten emerging sustainable technologies by the International Union of Pure and Applied Chemistry (IUPAC) in 2019.^[4]^ The advantages of organocatalysts include their lack of sensitivity to moisture and oxygen. Their broad availability, low cost, and low toxicity confers a huge direct benefit in the production of pharmaceutical intermediates when compared with (transition) metal catalysts. Critical aspects in their use are the high catalyst load needed and problems to recover these small molecule catalysts.

Nature also makes use of organocatalysts, but does so in a way which to a certain extent, solves the above‐mentioned issues. About half of all enzyme‐catalyzed reactions require cofactor(s) as part of their catalytic machinery.^[5]^ They are usually divided in coenzymes and prosthetic groups, with the latter ones being tightly bound to the protein. Some cofactors are responsible for the transfer of functional groups, such as SAM for methyl transfer or NAD(P)H for hydride transfer, others are electron donors or acceptors, and a few are catalysts. A description of all organic cofactors in nature as well as definitions, classification and their properties can be found in a review by Fischer et al.^[5]^

The cofactors acting as catalysts are embedded in proteins, whereby the protein scaffolds usually determine the reaction type, induce selectivity through specific binding pocket geometries and enhance the activity and efficiency. For physiological substrates, these binding pockets are evolutionary optimized for high affinity, steric as well as electrostatic properties and sometimes even provide part of the catalytic machinery. Usually, proteins can easily be immobilized and used in a batch‐ as well as in a flow‐reactor setup, thus solving the problems of catalyst reusability and recovery.

As the fourth wave of biocatalysis is approaching^[6]^ one of the foci is laid on the application of *de‐novo* designed enzymes or on enzymes carrying out new‐to‐nature reactions*. De‐novo* enzymes were successfully designed, and further improved via directed evolution. For purely computational/rational designs (examples are Retro‐Aldolase,^[7]^ Kemp‐Eliminase[^8^, ^9^] and Diels Alderase[^10^, ^11^]), the k_cat_/K_M_ was up to 10^2^ M^−1^ s^−1^ and was improved by extensive directed evolution approaches to rates in the range of 10^3^–10^5^ M^−1^ s^−1^.^[12]^ This is close to the rate of an'average’ enzyme (k_cat_/K_M_ of ∼10^5^ M^−1^ s^−1^), but far from the diffusion limit of 10^8^–10^9^ M^−1^ s^−1^.^[13]^

An alternative approach to extend the limited set of reactions carried out by natural enzymes is the utilization of artificial cofactors. This has been realized frequently in the last years using metal‐based catalysts.^[14]^ However, previous reviews have pointed out that organocatalytic cofactors should be considered more frequently when searching for new, non‐natural activities in enzymes.^[15]^

Here we focus on the emerging topic of catalytically active cofactors working in an organocatalytic fashion, as defined in the first sentence of this review. We highlight recent developments using artificial cofactors, based on organocatalysts, embedded in proteins and on non‐natural derivatives of natural organic (non‐metal) cofactors. We aim to provide a general overview and describe reaction types, substrates and employed catalysts in more detail.

## 2 Natural Organocatalytic Cofactors

**Figure 1:**
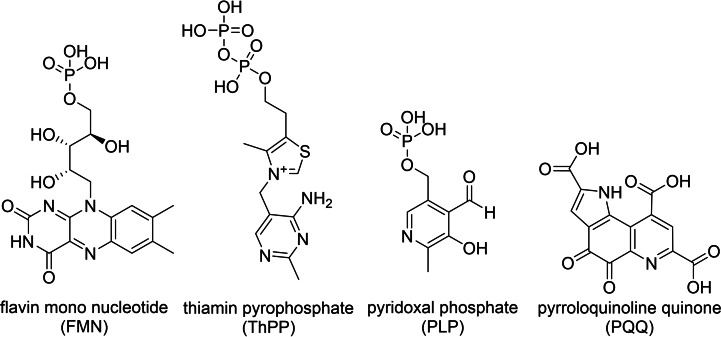
Examples of organocatalytic cofactors as used by natural occurring enzymes.

Nature uses several organocatalytic cofactors in enzymes as depicted in Figure 1. We will briefly introduce and highlight some selected features of them.

### 2.1 Flavins

**Figure 2:**
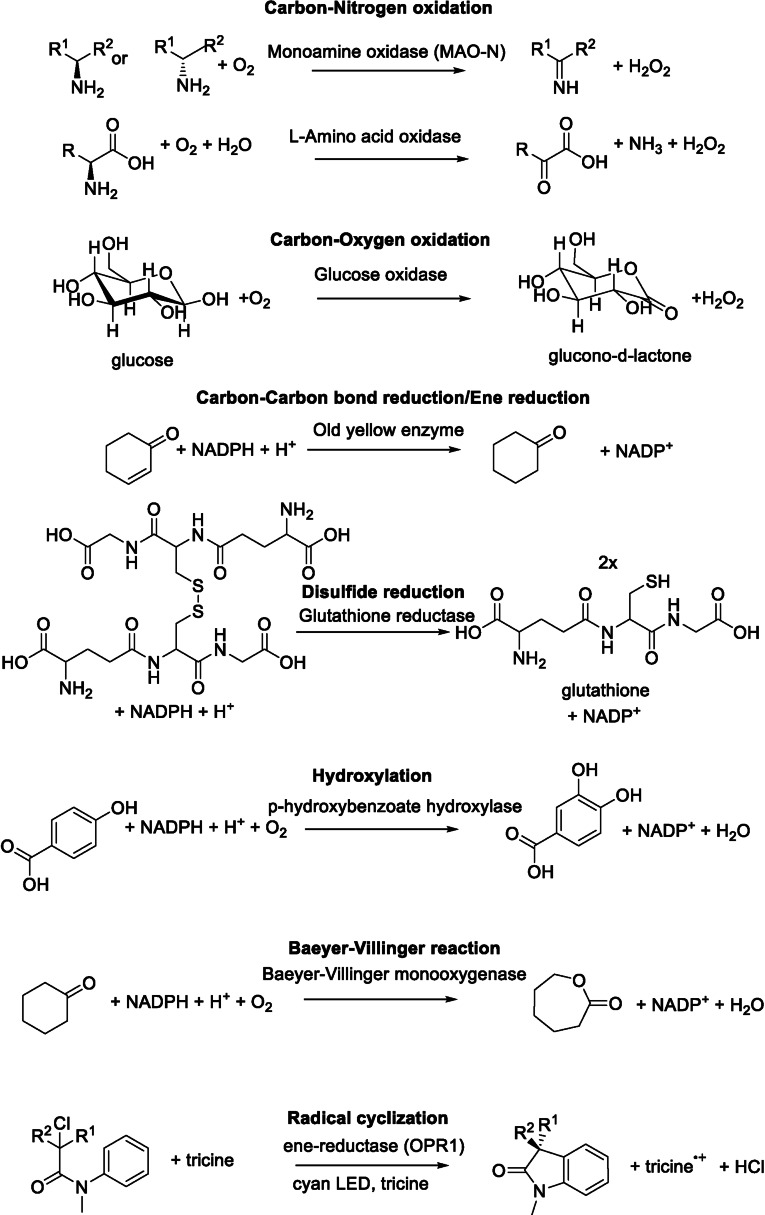
Selection of reactions carried out by various flavin‐dependent enzymes.

The most abundant and versatile cofactors are flavins. For most organisms it is estimated that 1‐3.5 % of their proteins bind flavin either covalently or non‐covalently.^[16]^ In nature, flavins occur either as flavin mononucleotide (FMN) or as flavin adenine dinucleotide (FAD) in most cases. Some organisms, however, use other derivatives. There are more than 20 different protein families that bind flavins, exhibiting for example TIM‐barrel and flavodoxin‐folds binding FMN and Rossman folds binding FAD. Flavins can perform reactions like (aromatic) (hydr‐)oxylations, demethylations or even (de‐)halogenations, which originates from their ability to participate in one‐ or two‐electron redox reactions or to form covalent adducts.[^17^, ^18^] They can also function as isomerases, lyases and ligases and are – via a rare catalytic species of the flavin – also responsible for reactions such as a Favorskii‐type rearrangement, a special cyclization reaction.^[19]^ They were reviewed by others,[^16^, ^18^, ^20^, ^21^] summarized in books^[17]^ and are an area of very active research. We provide a few examples of reactions carried out by flavin containing enzymes in Figure 2. To illustrate their full diversity would go beyond the scope of this review and is already documented elsewhere.[^16^, ^17^, ^18^, ^20^, ^21^]

In addition, we intend to highlight some natural derivatives of flavins. The redox‐active, oxygen‐insensitive 5‐deazaflavin cofactor (F_420_) was discovered in functionally versatile enzymes in a single phylum from both, bacteria (in methanogens), and archaea. Compared with FAD, it has a very low redox potential (−340 mV versus −220 mV). There was a very similar flavin derivative 8‐hydroxy‐5‐deazaflavin (F_o_) discovered, which in nature appears to be used for a single function only: as a light‐harvesting chromophore for DNA photolyases across the three domains of life. Both were reviewed recently.^[22]^

In 2015, a prenylated flavin (prFMN) was discovered as cofactor in an enzyme displaying activity for a non‐oxidative α,β‐unsaturated acid decarboxylation via a prFMN iminium species, which represents a new reaction mechanism for enzymes containing flavin(‐derivatives).[^23^, ^24^]

Another recently emerging topic using this cofactor concerns photocatalysis. Flavin‐dependent enzymes can carry out single‐electron transfers (SET) to or from organic substrates to create less commonly used open‐shell intermediates to access novel reaction types.[^25^, ^26^] Using light to photo‐reduce flavins was already observed as early as 1978 by Massey,^[27]^ who also stated that these systems can be used for SET. Recently this observation was rediscovered by the group of Hyster who reported the use of FMN‐dependent ene‐reductase and cyan LEDs used for photoreduction to catalyze a stereoselective redox neutral radical cyclization^[28]^ (Figure 2, bottom), reductive cyclization^[29]^ and the reduction of otherwise not converted substrates – acrylamides – through this single electron transfer.^[30]^ A general overview about the new reactivities of these well‐known enzymes following novel mechanisms was published in 2020.[^31^, ^32^]

In addition to their activity as cofactors in enzymes, flavins and flavin derivatives have now been explored as versatile “stand‐alone” organocatalysts[^33^, ^34^, ^35^] and have gained much of additional attention with the increasing interest in photocatalysis. As a result flavins are currently employed to act as photo‐redox catalysts for electron and hydrogen transfer reactions, which was reviewed very recently.^[36]^

### 2.2 Pyridoxal 5’‐phosphate

Pyridoxal 5’phosphate (PLP) or vitamin B6‐dependent enzymes exhibit an impressive catalytic versatility. Although the scope of PLP‐catalyzed reactions initially appears to be very diverse, there is a simple unifying catalytic principle; PLP easily forms Schiff bases with primary amino groups through its aldehyde moiety and stabilizes a negative charge that develops at the α‐carbon of the substrate in the transition state. The stabilization of the anion takes place through a delocalization of the negative charge through the π‐system of the cofactor, which is why PLP is often described as an electron sink. PLP can, similar to other naturally occurring catalytic cofactors, catalyze many of the described reactions slowly in the absence of any protein in buffered solutions. However, the surrounding amino acids in PLP‐dependent enzymes enhance this activity and enforce substrate selectivity and reaction type. Substrates, often amino acids, can react either on α‐, β‐, or the γ‐positions. Reactions on the α‐position include transamination, decarboxylation, and racemization. At the β‐ or γ‐position, elimination or replacement reactions are observed.

**Figure 3:**
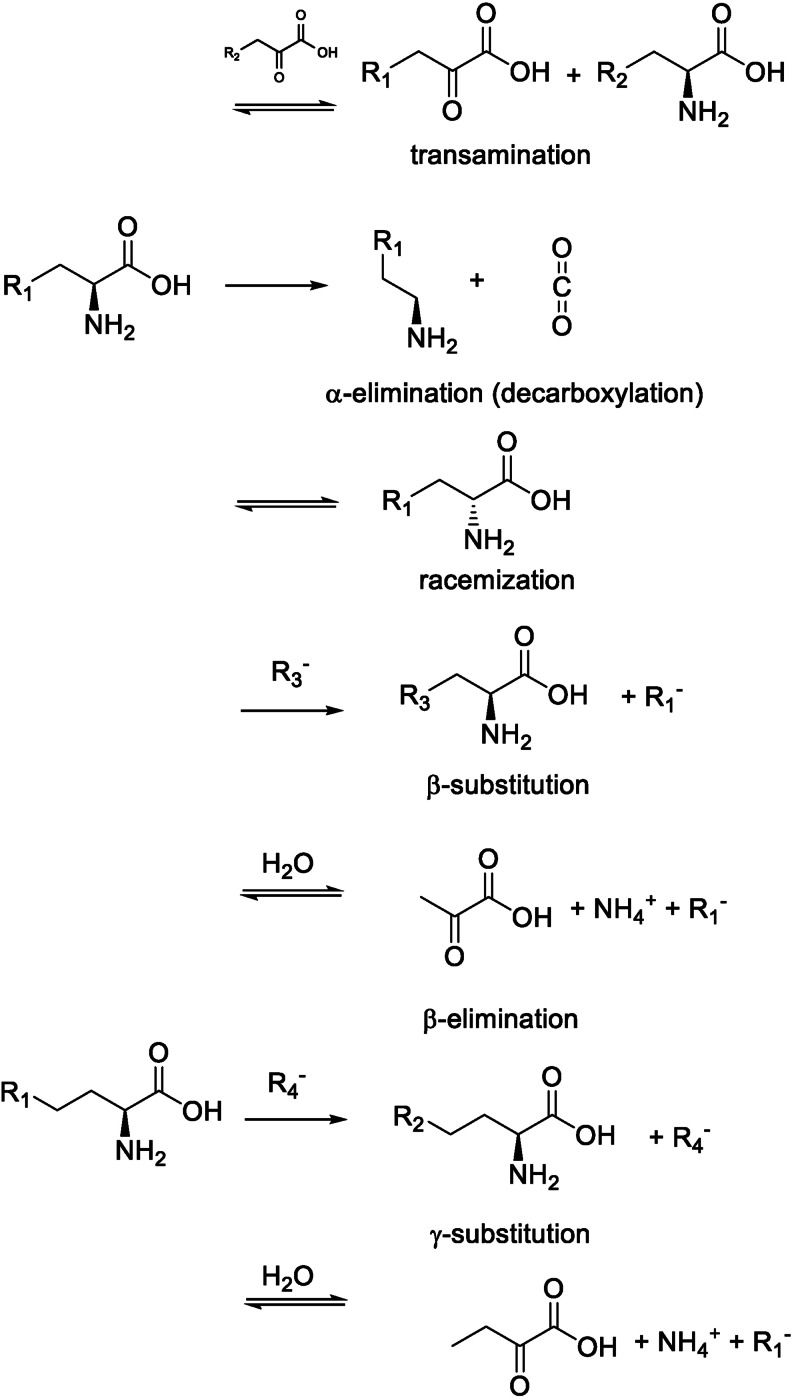
Selected reaction types carried out by the various PLP‐dependent enzymes.

Examples of each of these reactions are shown in Figure 3. PLP‐dependent enzymes are also known to catalyze several other reactions. Although not very common, a (retro‐)aldol^[37]^ and a (retro‐)Claisen^[38]^ reaction have been observed in some PLP‐dependent enzymes.

Structurally, these enzymes can be divided into seven different folds. Interestingly, there is no clear connection between folds/groups and specific activities. PLP‐dependent enzymes were reviewed in more detail several times[^38^, ^39^, ^40^, ^41^, ^42^] and are used for several biotechnological applications.^[43]^ The use of ω‐transaminases for the synthesis of chiral amines has to be particularly highlighted.[^44^, ^45^] An online database that provides information about all the 160 distinct functions of these enzymes and their genomic and structural information, as well as references to the respective studies is available.^[46]^

### 2.3 Thiamine pyrophosphate

Thiamine pyrophosphate (ThPP) serves as a cofactor in a number of enzymes found in many metabolic pathways. The catalyst is in principle a N‐heterocyclic carbene (NHC) – a powerful nucleophilic base catalyst (Lewis base). The active form of this catalyst – a carbene – would normally be unstable under physiological conditions, but is stabilized by the surrounding protein environment and was even observed in a structure of an ThPP‐dependent pyruvate oxidase.^[47]^ Catalysis using NHCs happens via a so‐called *umpolung*. Usually, electrophilic carbonyl carbon atoms become nucleophilic during the formation of the “Breslow” intermediate with the catalyst.^[48]^ In classical organic chemistry, these NHCs found broad application as organocatalysts to generate acyl anions, enolates, and homoenolates to form numerous high‐value complex products.^[49]^ Additionally, the NHCs are used as ligands for metal ions and *p*‐block elements^[50]^ and are applied in single electron transfers using photocatalysis as well.^[51]^

In nature, ThPP‐dependent enzymes catalyze a broad range of C−C, C−N, C−S, C−O bond forming and cleavage reactions. The known enzymes are collected in an online database containing more than 75 000 proteins^[52]^ and were reviewed extensively.[^53^, ^54^, ^55^] Again, the activity of the ThPP‐dependent enzymes relies mostly on the cofactor, while the protein modulates the catalytic properties and provides a binding pocket for the substrates.

Our intention is to highlight recent efforts for non‐physiological biocatalytic applications to catalyze stereoselective 1,2‐additions, like the benzoin addition to synthesize α‐hydroxy ketones, but also 1,4‐additions such as the Stetter reaction. These are promiscuous activities found in many members of this family. The following examples of their catalytic activities were reviewed previously in more detail.^[56]^

In the benzoin addition two aldehydes are added, leading to (aromatic) α‐hydroxyketones, benzoin or benzoin derivatives. The reactions can be carried out by promiscuous enzymes from the decarboxylase family. Most of the enzymes are very stereospecific for (*R*)‐enantiomers of the synthesized products – 2‐hydroxypropiophenone, phenylacetylcarbinol and benzoin. Using rational enzyme engineering, several variants were identified producing the corresponding (*S*)‐enantiomers with high selectivities. The authors could conclude that stereoselectivity in these enzymes is predominantly controlled by steric effects. More recently a rational “chimeric” pyruvate decarboxylase was used to produce (*S*)‐benzoin with enantioselectivities up to 99 %.^[57]^ Several interesting variations of the Benzoin reaction are described as well. For example the asymmetric intermolecular crossed aldehyde‐ketone Benzoin reaction^[58]^ in which ThPP is responsible for a decarboxylation reaction of pyruvate, yielding acetaldehyde, which reacts further with several ketones. There are also several examples of aliphatic‐aromatic aldehyde cross‐Benzoin reactions.^[59]^

For 1,4 additions – the Stetter reaction – three different ThPP‐dependent enzymes were identified, using aromatic as well as aliphatic α,β‐unsaturated ketones and pyruvate as substrates and yielding the corresponding 1,4‐carboligation products with moderate to good yields and selectivities.[^60^, ^61^] Noteworthy, as in the enzymatic transformations for the 1,2‐addition, the same enantiomer was always formed in excess.

ThPP‐dependent transketolases and variants of them were used to yield a range of aromatic and aliphatic ketodiol‐products with moderate to excellent stereoselectivities.^[56]^

ThPP or, in general, NHCs catalyzed reactions can quickly lead to a set of diverse and interesting products from simple starting materials and therefore this cofactor and derivatives might be interesting to be used with (re)designed proteins for the stereoselective synthesis of high‐value products or intermediates.

### 2.4 Other cofactors

The following compounds are described in order to provide a deeper overview of no‐metal natural cofactors. Those mentioned should not be considered as catalysts by themselves, but rather as co‐substrates (e. g. hydride or methyl‐group donors).

*S*‐Adenosyl methionine (SAM) is employed in nature as a methyl donor and degraded after the reaction. Thus, it cannot be reused in subsequent reactions.^[62]^ Recent efforts lead to interesting strategies to overcome this limitation for non‐radical SAM dependent reactions using a halide methyltransferase and methyl iodide to recycle SAM.^[63]^ Furthermore, a very broad family of enzymes employing a radical SAM mechanism is known. Often a 5‐deoxyadenosyl radical along with a [4Fe‐4S]^+^ cluster is employed for catalysis and a large variety of different reactions is observed, as reviewed in great detail by others.[^64^, ^65^, ^66^]

**Figure 4:**
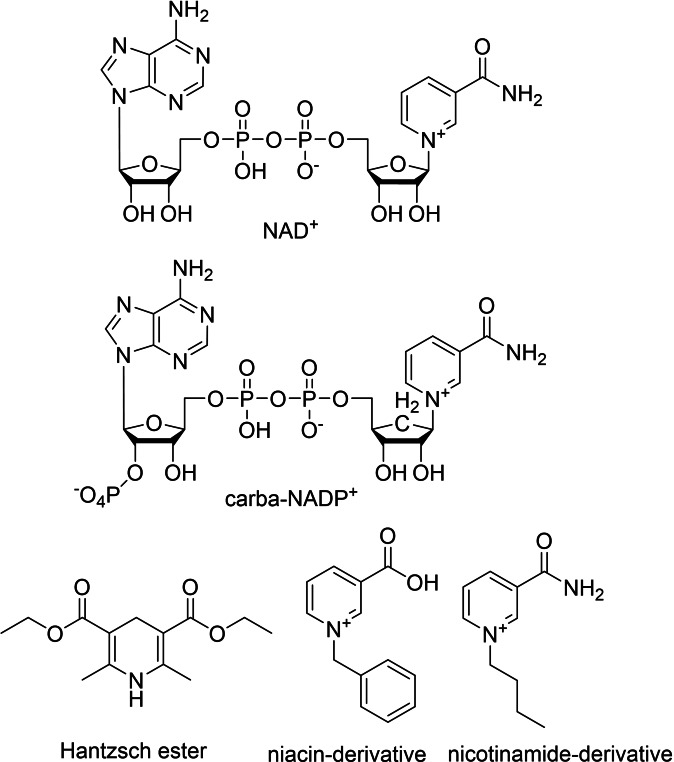
NAD and selected synthetic nicotinamide biomimetics.

Nicotinamide cofactors – nicotinamide adenosine dinucleotide (NAD(H)) or nicotinamide adenosine dinucleotide phosphate (NADP(H)) – are ubiquitous redox cofactors used as electron acceptors or electron donors. They are most commonly found in oxidoreductases, but can have other (non‐redox) functions as well. For the scope of this review the most interesting aspects regarding this cofactor are the synthetic nicotinamide cofactors developed recently and applied for enzymatic processes. Since they were reviewed extensively[^67^, ^68^, ^69^, ^70^, ^71^] we only depict some very selected examples[^72^, ^73^] in Figure 4 and Figure 11.

Finally, 4,5‐dihyro‐4,5‐dioxo‐1‐*H*‐pyrrolo[2,3‐*f*]quinoline‐2,7,9‐tricarboxylic acid (PQQ) or methoxatin is another redox cofactor. It is produced from a ribosomally derived post‐translationally modified peptide in prokaryotes, but can also be used by other organisms.^[74]^ PPQ‐dependent enzymes are usually dehydrogenases that catalyze the oxidations of a variety of alcohols, aldehydes and sugars and have been reported to occur only in 8‐bladed β‐propeller folds.[^75^, ^76^, ^77^] They do not use NAD(P)^+^ as electron acceptor. Instead, in their oxidative half‐reaction, the soluble PQQ‐dependent dehydrogenases donate electrons to redox proteins like cytochrome *c* or to electron acceptors like Fe^3+^. This was shown by using a PQQ‐dependent dehydrogenase to convert a racemic alcohol to a chiral amine in a cascade reaction.^[78]^ Membrane‐bound PQQ‐dependent dehydrogenases donate electrons to ubiquinone. In general, this cofactor is the most recent and least understood of all the mentioned ones in this chapter.

## 3 Non‐natural Derivatives of Natural Organocatalytic Cofactors

### 3.1 Flavin derivatives

There is a large number of flavin derivatives that are mostly used to catalyze oxidations. The flavin cofactor is easily removed from natural enzymes and can subsequently be substituted with a derivative. In addition, they can also serve as stand‐alone catalysts. When used in this fashion, a higher catalyst load is usually needed, and racemic products are gained. There are several reviews covering this topic from a chemists perspective.[^34^, ^79^, ^80^] Here, we focus only on some examples where a flavin derivative was used as a cofactor in a protein scaffold.

**Figure 5:**
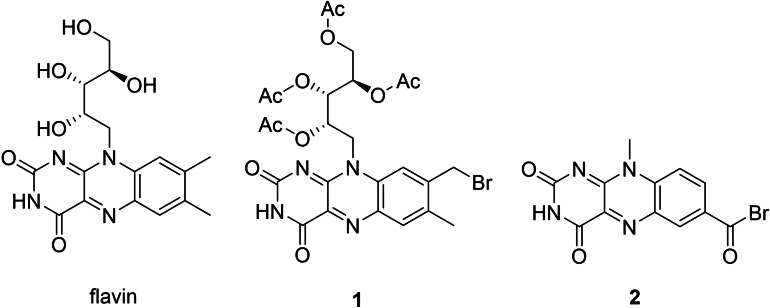
Flavin derivatives used as cofactors; **1** and **2** were covalently bound to the cysteine of the active site of papain.

In the 1970s, the first non‐natural derivatives of flavin cofactors **1** and **2** (Figure 5) were introduced to papain by covalent attachment to the activated cysteine in the original active site of this protease.^[81]^ The semi‐synthetic enzyme with cofactor **2** (Figure 5) was able to oxidize N‐benzyl‐1,4‐dihydronicotinamide with k_cat_/K_M_ similar to other flavin‐oxidases and a 50 fold rate acceleration compared to the bare cofactor.

Several other flavin derivatives were synthesized as spectral, chemical, and mechanistic probes.^[82]^ An interesting 8‐CN flavin with a high oxidation‐reduction potential was synthesized and characterized using several apo‐proteins.^[83]^ Substitution of the flavin cofactor in old yellow enzyme (OYE) with this derivative lead to a Desaturase, yielding α‐β unsaturated aldehydes like cinnamaldehyde.^[84]^

**Figure 6:**
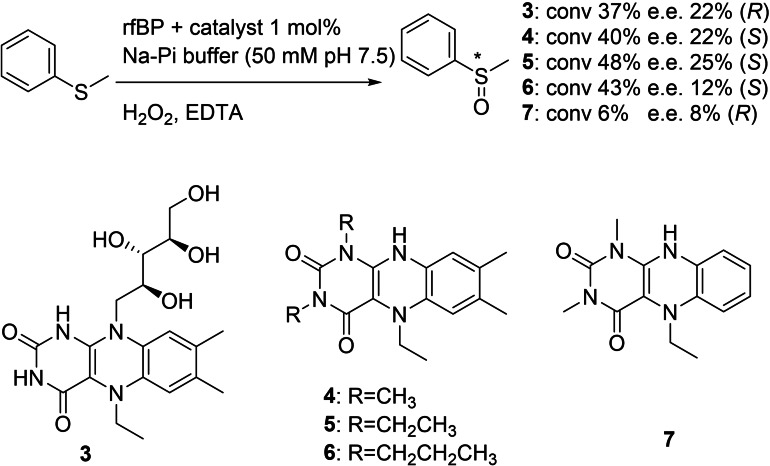
Flavin derivatives bound non‐covalently as cofactors to a riboflavin binding protein for stereoselective sulfoxidations.

More recently, flavin derivatives were used in combination with a riboflavin binding protein from *Gallus gallus* ^[80]^ to generate peroxide‐driven monooxygenases.^[57]^ The artificial enzymes were able to carry out enantioselective sulfoxidations on a range of sulfides. An example is shown in Figure 6.

### 3.2 PLP derivatives

**Figure 7:**
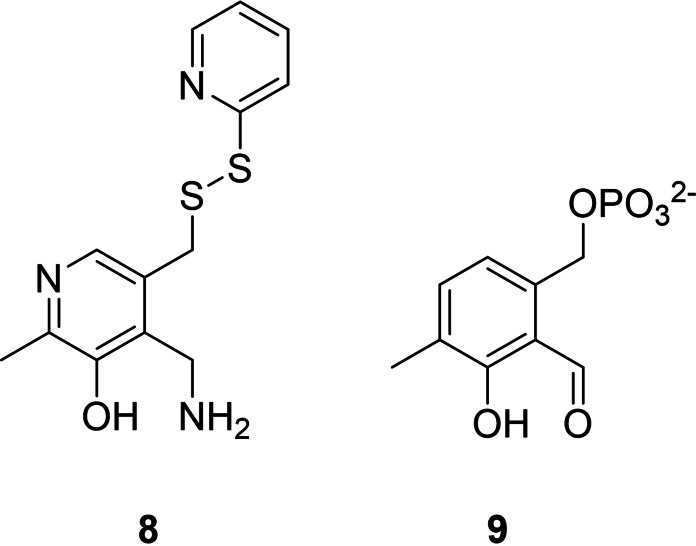
Pyridoxal‐derivatives used in fatty acid binding proteins as cofactor and deazapyridoxal 5′‐phosphate.

**Figure 8:**
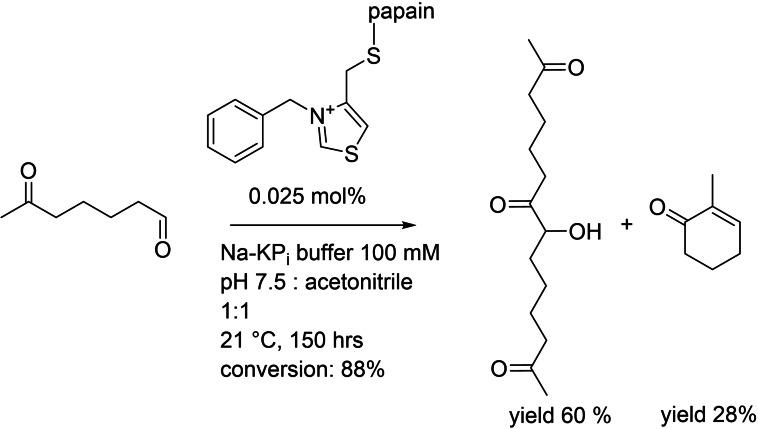
Thialzolium modified papain employed for C−C bond formations.

There are also reports of proteins harboring pyridoxal‐groups as non‐natural cofactors. For example a 6‐fluoro pyridoxal phosphate bound to a phosphorylase leads to a lower activity of this enzyme compared to natural PLP, due to a lower basicity of the pyridine nitrogen.^[85]^ Distefano and co‐workers attached a pyridoxal derivative (Figure 7, **8**) to a suited cysteine (C117) in the adipocyte lipid binding protein via a disulfide bond.^[86]^ They performed reductive aminations on various substrates. The protein around the catalyst decreased the initial rate of the reductive amination of pyruvate 1.8‐fold compared to the bare cofactor, but lead to the stereoselective formation of amino acids like L‐valine with an enantiomeric excess of 94 %, using pyruvate as amine‐donor. In a follow‐up study they used the same pyridoxal derivative (Figure 7, **8**) in a V60C variant of intestinal fatty acid binding protein as attachment point for the cofactor.^[87]^ This protein‐cofactor complex showed a 62‐fold rate enhancement compared to the bare cofactor in solution, due to a much better K_M_ for the substrate. With glutamate as substrate, several racemic amino acids were used as amine donor. The formation of L‐glutamic acid with an e.e. up to 94 % was possible. The disadvantage of the system is the modest turnover numbers achieved within 24 hours.

**Figure 9:**
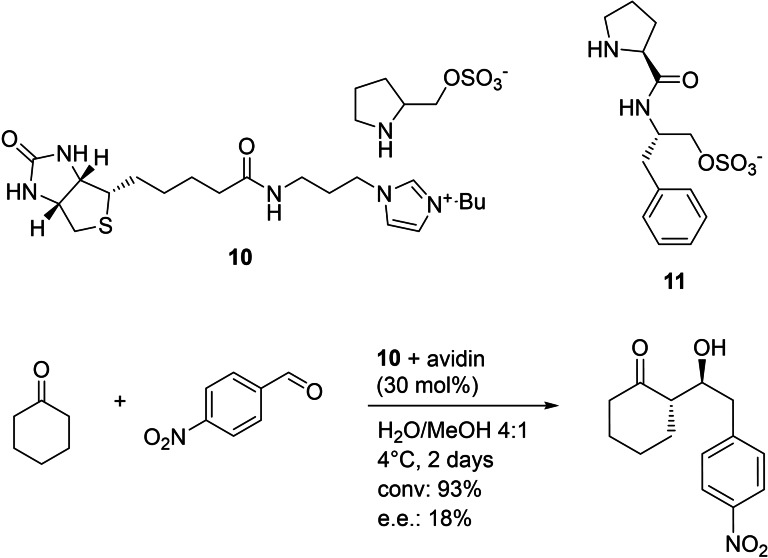
Imidazolium salts used in a biotin‐streptavidin system as artificial cofactor for a stereoselective Aldol reaction.

Another example for an employed PLP derivative is deazapyridoxal 5’‐phosphate (Figure 7, **9**).^[88]^ Although more in the context of a mechanistic study, the authors could show that the PLP‐derivative **9** can be applied as bio‐orthogonal cofactor for an alanine racemase and O‐acetylserine sulfhydrylase, but with 700‐ and 250‐fold reduced activity compared to the natural cofactor, PLP. For amino transferases (e. g. aspartate aminotransferase), the formation of the carbocationic intermediate is necessary. This is not possible with **9** and lead to ∼10^8^ fold decreased activity using this non‐natural cofactor.

Lastly, several PLP derivatives with functional groups enabling click chemistry were developed for proteomics as cofactor probes.^[89]^ They might be used to develop new PLP dependent enzymes, where the cofactors are attached using non‐canonical amino acids via the aforementioned click chemistry.

### 3.3 ThPP derivatives

The catalytic moiety of ThPP was used in 1993 by modification of the active site cysteine from papain with a *N*‐benzyl‐2‐bromomethylthiazolium salt leading to C−C bond formations using 6‐oxoheptanal as substrate.^[90]^ Two reactions were observed: a dimerization (major) and a cyclization reaction (minor) of the substrate (Figure 8). This artificial enzyme is roughly three times more active than the catalyst itself.

## 4 Non‐natural (Artificial) Organocatalytic Cofactors

Recently, several non‐natural organocatalytic cofactors were developed and used mostly for C−C bond formation. Interestingly, all of these artificial enzymes use the well‐known (strept)avidin‐biotin system. This system was used for the first time in the 1970s by Whitesides^[91]^ and since 2000 saw excessive and very successful use by Ward and co‐workers. They introduced non‐natural metal based cofactors (complexes) into a protein environment, leading to stereoselective catalysts after rational protein design around the binding pocket and optimization of the catalyst, as reviewed elsewhere.^[92]^ Biotin, the natural ligand of streptavidin, can be easily modified on a carboxylic acid group, pointing towards a grove at the interface of its homotetramer. Since biotin binds to (strept)avidin with a K_d_ of ∼10^−15^ M, this interaction can almost be considered covalent.

One of the first examples reported was the use of a biotinylated imidazolium salt **10** for a stereoselective aldol reaction (Figure 9).^[93]^ The authors claim that neither the biotinylated catalyst nor avidin was able to catalyze the reactions under the reported reaction conditions alone, but the combination of both lead to conversions up to 99 %. Low chirality was induced using organic cosolvents. An e.e. up to 70 % was possible using an ionic liquid (1‐butyl‐3‐methylimidazolium/Br pH 3.0) as cosolvent, but with lower conversion (43 %). The catalyst was improved via variation of the anion salt (Figure 9, **11**) leading to an e.e. of 80 % in the model reaction.^[94]^

**Figure 10:**
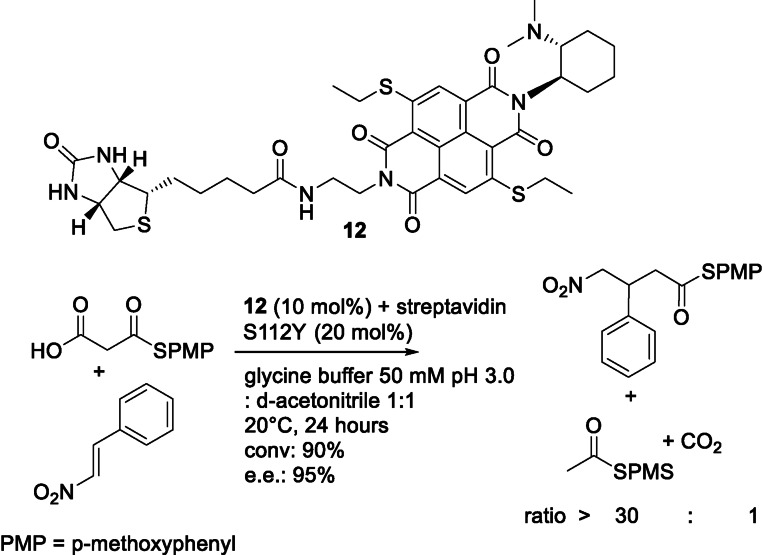
Anion‐π catalyzed carbon‐carbon bond formation of an enolate acceptor a malonic acid half thioester.

Ward, Matile and co‐workers used the streptavidin‐biotin system to anchor a naphthalenediimide with a base substituent.^[95]^ This catalyst is able to facilitate a rare anion‐π interaction that does not occur in nature to catalyze a C−C bond formation – a Michael addition – via the addition of malonic acid half thioesters to enolate acceptors. Five different catalysts were used in eleven different streptavidin variants. The best variant (Figure 10) exceeds the performance of conventional organocatalysts yielding a conversion of 90 % with an enantiomeric excess of 95 %. This is a remarkable example of a non‐natural reaction catalyzed by a non‐natural cofactor in a protein variant leading to extraordinary results in terms of stereoselectivity and conversion. Neither the catalyst alone nor the protein scaffold (streptavidin) were able to catalyze the reaction when used separately.

**Figure 11:**
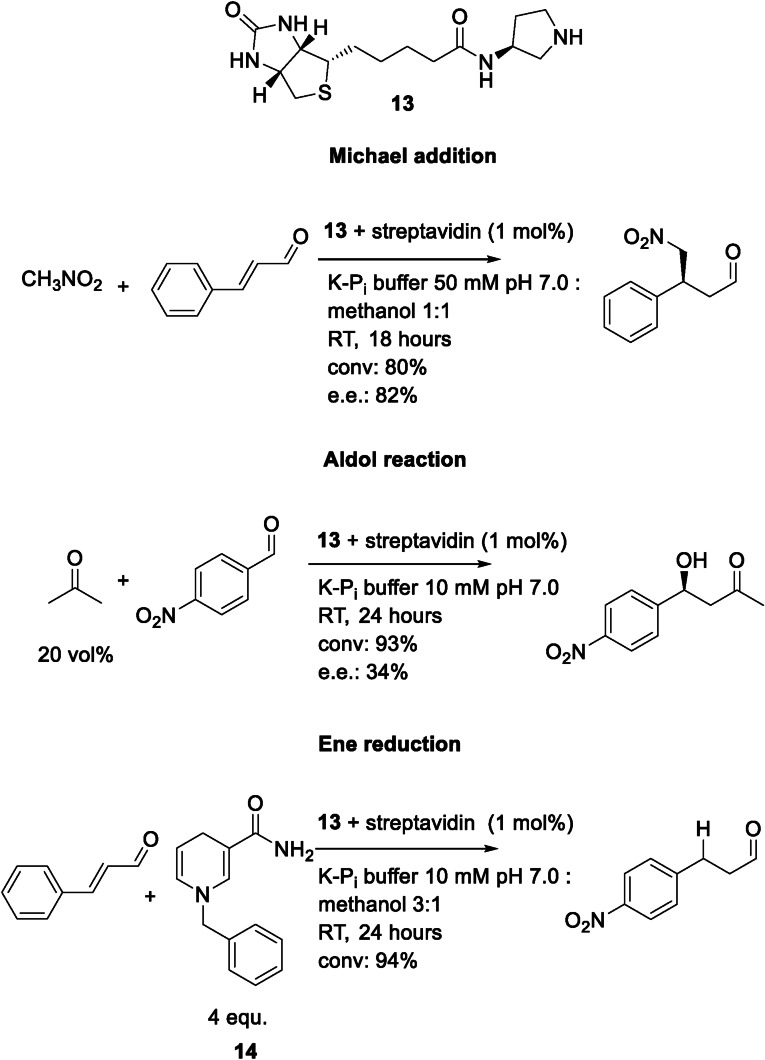
The variety of reactions catalyzed by an artificial pyrrolidin‐biotin catalyst with streptavidin as protein scaffold.

The group of Luk synthesized biotinylated 4‐imidazolidinone, proline and pyrrolidine derivatives as catalysts for the formation of an imine as the reactive intermediate and employed them as artificial cofactors in commercially available streptavidin for Michael additions.^[96]^ The reactions can be catalyzed by streptavidin as well as the biotinylated catalysts alone, but lead to a racemic product and 7 % yield in both cases, using only the most promising cofactor **13**, in a buffered system. After reaction optimizations, which lead to the addition of 50 % v/v co‐solvent, they achieved a good yield (80 %) and stereoselectivity with their model substrate and **13** as cofactor bound to streptavidin (Figure 11). A small substrate scope study was performed using five different aldehydes and a ketone. However, the latter was only marginally converted (<5 %). In a follow‐up work, they employed several variants of streptavidin (single‐point variants as well as dimeric and monomeric variants) to increase conversion and selectivity further. An L124W variant was able to increase yields up to 92 % displaying the similar selectivities.^[97]^ Using **13**, an aldol reaction was also carried out, using four different streptavidin variants.^[98]^ However, no improvement compared to the wildtype protein for the low stereoselectivity of the aldol reaction could be achieved. The conversion was up to 93 %, but the cofactor itself was able to convert 36 % of the substrates to product. The same artificial cofactor (**13**) as catalyst was used for a third reaction using streptavidin as protein scaffold – the reduction of an α,β unsaturated aldehyde using a dihydrobenzyl nicotinamide (BNAH) **14** as hydride donor.^[99]^ For optimization they used monomeric as well as tetrameric streptavidin with various hydride donors. As additional substrates, a range of para‐substituted cinnamaldehyde analogs were tested and all of them were reduced. In this case, the protein was not inducing any chirality, but it was enhancing the activity. The use of the cofactor **13** and the hydride donor **14** lead to only 30 % conversion compared to the catalyst embedded in streptavidin.

**Figure 12:**
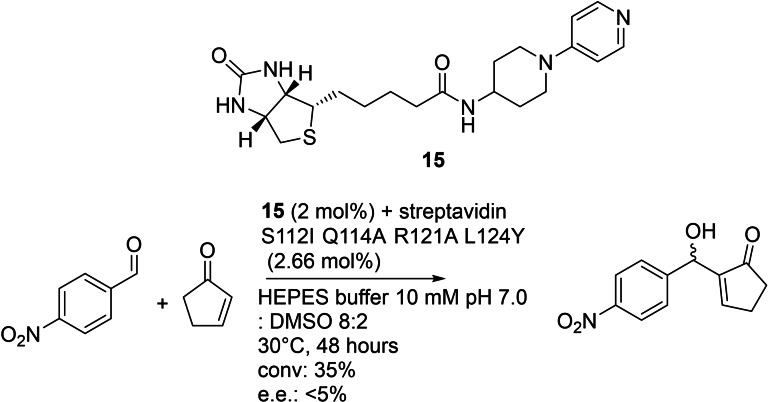
Baylis‐Hillman reaction catalyzed by a biotinylated DMAP‐derivative and streptavidin variants as protein host.

In another study, a biotinylated nucleophilic catalyst 4‐(4‐aminopiperidino)pyridine (Figure 12, **15**), a dimethylaminopyridine derivative, with streptavidin as protein host, was used to catalyze a Baylis–Hillman reaction.^[100]^ Here only one cofactor was synthesized. The reactivity varied and was tuned by the exchange of several amino acids at the active site of streptavidin. Finally, a cofactor **15** bound to a quadruple variant of streptavidin yielded in 35 % conversion for the model substrates, but with no enantioselectivity. An increase in size of the aldehyde by the use of isatin instead of *p*‐nitrobenzaldehyde as model substrate, did not lead to a detectable stereoselectivity either. The cofactor itself showed only minor activity (∼1 % yield), but streptavidin as protein without any further modifications was able to catalyze the reaction with yields up to 11 %. The combination of both, protein, and cofactor lead to a 3‐fold increase in conversion.

We have provided an overview of recent developments in the design of new enzymes with organocatalytic cofactors. The natural organocatalytic cofactors themselves span a very interesting range of reactions in terms of reactivity as well as products formed. We illustrated how derivatives of natural cofactors can be used for new reactions and highlighted how they can (attached to protein scaffolds originally not able to accommodate these catalysts) be the basis for new selectivities. Some catalysts, as for example the NHC catalyst ThPP, are even able to catalyze reactions not necessarily exploited by nature and are therefore interesting starting points for the development of non‐natural new enzymatic activities in the future.

Furthermore, we reported showcases in which new‐to‐nature organocatalysts were embedded in proteins as artificial cofactors to achieve better activities and/or selectivities in the used buffered reaction systems. Since many of these cover carbon‐carbon bond forming reactions, which are desired in a selective manner for many synthetic routes, we expect more will come soon in this direction.

We illustrate how cofactor‐dependent enzymes can be exploited to promote reactions first established in organic chemistry with related chemical catalysts. The ability of proteins to enhance the reactivity of the used cofactors and control selectivity by the surrounding protein scaffold together with the ability to optimize non‐natural reactivity via directed evolution, promises to yield catalysts for transformations that have no biological counterparts. This has already been shown for many artificial metal‐based cofactors. Advantages of organocatalyst‐based cofactors over metal‐based ones are their lower complexity, ready accessibility, smaller size and lower toxicity than some (rare‐earth) metal complexes.

From the perspective of an enzyme designer, it is advantageous to use an already active catalyst as starting point to gain new reactivities compared to full *de‐novo* design approaches. When designing an enzyme *de‐novo*, gaining initial activity is often hard to achieve. At the beginning of such an endeavor, many different variants must be screened to find some with minimal activities. The starting activity is already given, when using an (organo)catalyst, and can be improved by designing a selective, reaction‐rate enhancing binding pocket within the protein. This can be achieved by means of computational design or directed evolution. One might argue here that those cofactors need a proper designed binding pocket. However, many examples of designed ligand binding proteins or anchoring of catalysts via various methods to a protein of choice show that this is more readily achievable than the creation of an enzymatic active site from scratch. Another point of concern is the reactivity of the catalysts in buffered systems. Most of them are initially developed and tested in organic solvents, but as reviewed recently,^[101]^ a growing number of organocatalysts employed in aqueous systems exists for a variety of reaction types. Some have already been employed as artificial cofactors, while some others have not yet been used for the purpose, although they have potential as such.

With an approach, combining small‐molecule catalysis, synthetic chemistry, enzymology, computational chemistry, protein design and directed evolution, a fascinating interdisciplinary research field is developing, leading to new enzymes with non‐natural reactivities and combining advantages of biocatalysis with the world of organocatalysis.

Since all examples of the non‐natural organocatalytic cofactors are less than seven years old, we are expecting that more will come in the near future.

## Conflict of interest

The authors declare no conflict of interest.

## Biographical Information

*Horst Lechner studied chemistry and biochemistry at the University of Graz (Austria) where he received his PhD in biocatalysis in 2015 in the group of Prof. Kroutil. He subsequently moved to Prof. Höcker's laboratory (University of Bayreuth, Germany) as postdoctoral fellow. He is currently a postdoc in Prof. G. Oberdorfer's laboratory at the Graz University of Technology (Austria) with interests in (re)designing enzymes and protein/ligand interactions*.

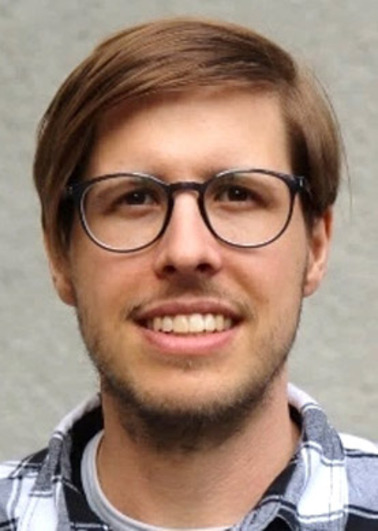

## Biographical Information

*Gustav Oberdorfer obtained his PhD in biochemistry and molecular biology from the University of Graz in 2011. He then joined the laboratory of Prof. David Baker (University of Washington, United States) as a postdoctoral fellow in 2012. In 2020 he joined the faculty of the Institute of Biochemistry at the Graz University of Technology as an Assistant Professor. The major research interests of his group are in computational protein design, with a focus on enzyme and small‐molecule binder design*.